# CyTOF reveals platelet subtype changes predicting the efficacy of combined immunotherapy and targeted therapy in liver cancer

**DOI:** 10.3389/fimmu.2025.1538652

**Published:** 2025-05-27

**Authors:** Wenjing Wang, Dan Liu, Lilin Wang, Maimaitijiang Wubuli Aishan, Li Chen, Sujun Zheng, Junfeng Lu

**Affiliations:** ^1^ Beijing Institute of Hepatology, Beijing YouAn Hospital, Capital Medical University, Beijing, China; ^2^ First Department of Liver Disease, Beijing YouAn Hospital, Capital Medical University, Beijing, China; ^3^ Department of Liver Disease, Hotan District Infectious Disease Specialist Hospital, Hotan, Xinjiang Autonomous Region, China

**Keywords:** liver cancer, platelets, CyTOF, combined immunotherapy and targeted therapy, CD29+

## Abstract

**Introduction:**

Immune checkpoint inhibitors combined with angiogenesis inhibitors are currently the first-line treatment for liver cancer. However, some patients still exhibit poor therapeutic outcomes. Platelets, as a critical component of blood, play a significant role in liver cancer progression by influencing angiogenesis and the tumor immune microenvironment.

**Methods:**

In our study, we utilized mass cytometry (CyTOF) to analyze surface proteins on platelets in the plasma of 23 liver cancer patients before and after receiving combined immunotherapy and targeted therapy. Patients were grouped based on treatment efficacy to compare platelet subpopulation differences.

**Results:**

We observed that CD107a+ and CD62P+ platelet subpopulations were reduced in liver cancer patients. In the progressive disease (PD) group, the CD29+ platelet subpopulation was elevated compared to other groups. Notably, this subpopulation decreased with tumor remission and increased with tumor progression.

**Discussion:**

Our findings highlight the heterogeneity of platelets in liver cancer patients and suggest that the CD29+ platelet subpopulation may serve as a predictive biomarker for the efficacy of combined immunotherapy and targeted therapy. Additionally, CD29+ platelets could represent a potential therapeutic target in future research.

## Graphical Abstract

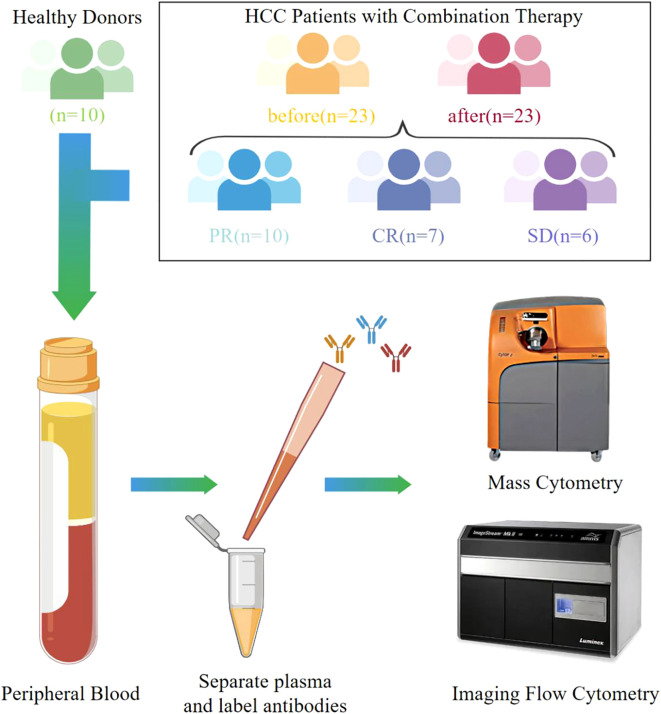

## Introduction

Liver cancer is the sixth most common cancer worldwide and the third leading cause of cancer-related deaths, following lung cancer and colorectal cancer (1). Current systemic treatments for liver cancer include anti-angiogenic targeted therapy and immunotherapy (2). Due to the liver’s high blood flow and the overexpression of pro-angiogenic factors in the immune microenvironment of hepatocellular carcinoma (HCC), HCC is considered a highly angiogenic tumor (3). Anti-angiogenic targeted therapy has shown good efficacy in treating liver cancer (4). In recent years, with the advancement of clinical trials, the combination of immune checkpoint inhibitors and anti-angiogenic targeted therapy has also become a first-line treatment for liver cancer (5).

During the process of combined immunotherapy and targeted therapy, immune inhibitors block immune checkpoints, allowing T cells to more effectively recognize and attack tumor cells (6). At the same time, anti-angiogenic targeted therapy reduces tumor angiogenesis and blood supply by inhibiting downstream signals of angiogenesis, thereby suppressing tumor growth (7). Targeted therapy can enhance the sensitivity of tumors to immunotherapy, and the combination of immunotherapy and targeted therapy can also reduce tumor resistance, resulting in a synergistic anti-tumor effect (8).

Platelets are small cell fragments derived from megakaryocytes that play a crucial role in blood clotting and wound healing (9). When blood vessels are damaged, platelets can recognize the injury site, aggregate to form a hemostatic plug, and further promote the activation of clotting factors (10). Platelets are also involved in inflammatory responses and immune regulation, interacting in complex ways with various immune cells (11). Additionally, platelets can release angiogenesis-related growth factors, regulating the formation of blood vessels (12). There is existing literature reporting that platelets also play an important role in tumors, with a general consensus that platelets and platelet receptors can influence cancer metastasis (13, 14).

CyTOF (Cytometry by Time-Of-Flight) can detect protein markers on cells, providing new insights for analyzing cellular heterogeneity, and is widely used in various fields (15). Different types of platelets exhibit distinct functions (16). While there have been studies using CyTOF to analyze platelets, research specifically focused on liver cancer is limited (17, 18).

Given that platelets play roles in both immune regulation and angiogenesis, and the liver is one of the most blood-rich organs in the body, we aimed to explore the function and changes of platelets in combined immunotherapy and targeted therapy for liver cancer in this study. We used CyTOF technology to detect platelets in plasma, distinguishing them based on different surface proteins and analyzing the differences. We found significant differences in platelets between liver cancer patients and healthy donors, as well as among different prognostic groups of liver cancer patients. Activated platelet subpopulations were reduced in liver cancer patients, and CD29+ platelets could serve as markers of poor prognosis for combined immunotherapy and targeted therapy in liver cancer.

## Materials and methods

### Agents

Some antibodies were labeled with commercially available metal-conjugated antibodies, while others were labeled using custom-conjugated metal-labeled antibodies according to the metal antibody labeling method provided by Standard Biotools. All antibody information is presented in **Supplementary Table 1**. The Maxpar X8 Multi-Metal Labeling Kit, EQ Four Element Calibration Beads, and FixI were purchased from Standard Biotools. DPBS was purchased from Corning; EDTA anticoagulant tubes from BD; TCEP from Thermo; antibody storage solution from Candor Bioscience; and 3 k and 50 k filters from Amicon Ultra.

### Patients

This study included 23 patients with hepatocellular carcinoma undergoing anti-angiogenic agent therapy in combination with immune checkpoint inhibitors at Beijing You’an Hospital. All patient information is presented in **Supplementary Table 2**. These patients were treated with Sindilizumab as an immune checkpoint inhibitor, administered at a fixed dose of 200 mg via intravenous injection every three weeks. Lenvatinib or Bevacizumab was used as an anti-angiogenic agent, with Lenvatinib administered orally at 8 mg to 12 mg per day, or Bevacizumab administered intravenously at a dose of 15 mg/kg every three weeks. The treatment duration was more than 12 weeks. Based on treatment efficacy, patients were categorized into PD, SD, and PR groups. The PD group comprised patients with a lesion diameter increase of more than 20% or the emergence of new lesions. The SD group included patients with a lesion diameter reduction of less than 30%. The PR group consisted of patients with a lesion diameter reduction exceeding 30%, sustained for at least four weeks. Tumor size before and after treatment, along with patient grouping details, is presented in **Supplementary Table 3**. Inclusion criteria included age over 18, clinically or pathologically confirmed primary liver cancer (Guidelines for the Management of Primary Liver Cancer v2022), stage II or III according to the diagnostic staging of hepatocellular carcinoma in China, and ineligibility for surgical treatment. Child-Pugh score ≤ 10, with no serious cardiac, pulmonary, or renal disease, and a life expectancy of at least 3 months. Eastern Cooperative Oncology Group performance status of 0 or 1, with no prior systemic therapy. All patients provided written informed consent to participate based on the principles of the Declaration of Helsinki.

### Metal antibody labeling

First, 95 μl of L-Buffer was added to the X8-polymer tube and mixed until the polymer was completely dissolved. Then, 5 μl of a 50 mM lanthanide metal element solution was added, mixed, and incubated in a water bath at 37°C for 30 minutes. A total of 100 μg of antibody was added to a 50k filter, and R-buffer was added to reach a total volume of 400 μl. The sample was centrifuged at 12,000 g at room temperature for 10 minutes. TCEP was diluted in R-buffer to a final concentration of 4 mM (1:100). A volume of 200 μl of L-Buffer was added to a 3k filter, and the metal-polymer mixture was transferred to the 3k filter. The sample was then centrifuged at 12,000 g at room temperature for 30 minutes. Next, 100 μl of the 4 mM TCEP solution was added to the 50k filter containing the antibody, mixed, and immediately incubated at 37°C for 30 minutes. The waste in the 3k filter collection tube was discarded, 400 μl of C-buffer was added, and the sample was centrifuged at 12,000 g at room temperature for 30 minutes. Then, 300 μl of C-buffer was added to the 50k filter, and any remaining antibody was gently aspirated from the tube walls. The sample was centrifuged at 12,000 g at room temperature for 10 minutes, the waste in the 50k filter collection tube was discarded, and 400 μl of C-buffer was added for a second wash. The sample was centrifuged again at 12,000 g at room temperature for 10 minutes. After centrifugation, the 3k filter was removed, followed by the 50k filter. The correspondence between each antibody and metal was confirmed. A volume of 60 μl of C-buffer was added, and all liquid was transferred to the 50k filter, then gently mixed by pipetting. The sample was incubated in a water bath at 37°C for 90 minutes. After incubation, the 50k filter was removed from the water bath, and 300 μl of W-buffer was added. The sample was centrifuged at 12,000 g at room temperature for 10 minutes, the waste in the collection tube was discarded, and the filter was washed three times with W-buffer. After the final wash, 100 μl of antibody storage solution was added, and the filter was inverted into another collection tube. The sample was centrifuged at 1,000 g at room temperature for 2 minutes to collect the antibody. Finally, the antibody was stored at 4°C for later use.

### Platelet-rich plasma separation

Blood samples were collected before treatment and a few hours afterward, while samples from 10 healthy donors served as a control group. After centrifugation, the plasma layer was transferred to 0.5 ml polypropylene tubes and frozen at −80°C. The donors had not used antiplatelet drugs within the past two weeks. Blood was collected from the elbow vein using 5% EDTA anticoagulant tubes and centrifuged at 200 g for 10 minutes. The upper layer containing platelet-rich plasma was then collected.

### Labeling of platelets with metal-conjugated antibodies

Metal-conjugated antibodies were incubated with 200 μl of platelet-rich plasma at room temperature for 30 minutes. Subsequently, 1 ml of DPBS was added, followed by centrifugation. Next, 1 ml of Fix I fixative solution was added, mixed, and centrifuged at 800 g for 5 minutes. After three washes with double-distilled water, the platelets were mixed with 10% EQ beads and filtered through a 40 μm filter before analysis using Helios mass cytometry. For each sample, 100,000 events were collected.

### CyTOF data analysis

The CyTOF data analysis process began with sample preprocessing using CyTOF software version 7.0. Raw FCS files were normalized using EQ Four Element Calibration Beads, resulting in standardized FCS files. These standardized data were then uploaded to the Cytobank platform (https://www.Cytobank.org) for further analysis and processing. CD41a+ platelets were isolated by applying gating, while simultaneously removing beads and debris. The gated data were subsequently exported for more comprehensive analysis using R (https://bioconductor.org/packages/cytofkit/). To enhance data visualization and understanding, Phenograph clustering and T-SNE (t-distributed Stochastic Neighbor Embedding) were used for dimensionality reduction. Additionally, statistical analysis was performed to assess clustering results and protein expression for each sample. Furthermore, Sangbox (http://www.sangerbox.com/login.html) was utilized for correlation analysis, evaluating the impact of different cell clusters and protein expression on survival outcomes.

## Result

### Differences in platelets between liver cancer patients and healthy donors

**Figure 1:**
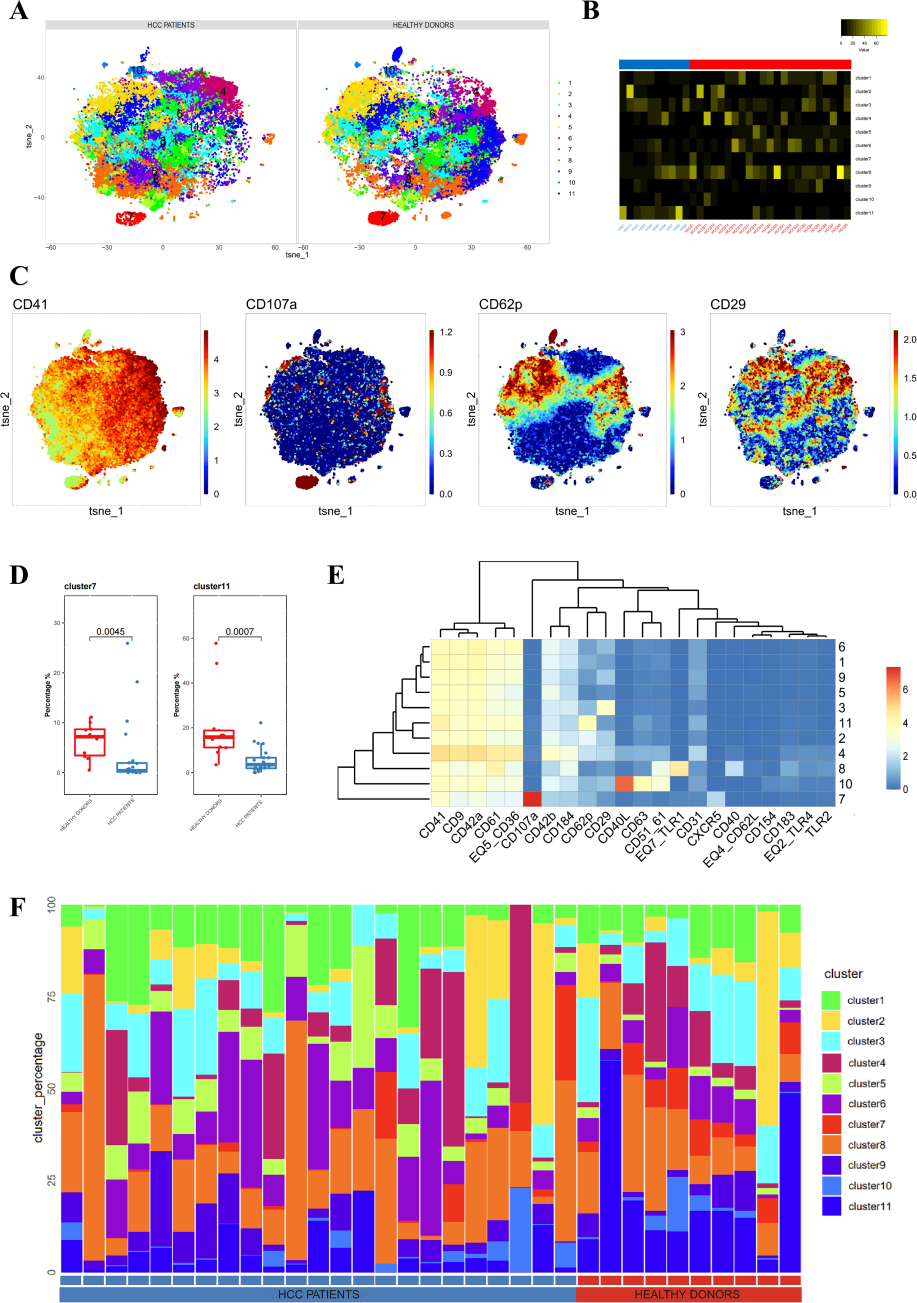
Differences in platelets between untreated liver cancer patients and healthy donors. **(A)** CyTOF analysis showing the differences in platelets between untreated liver cancer patients and healthy donors. t-SNE clustering based on protein expression levels in different groups (NORMAL group for healthy donors, BEFORE group for liver cancer patients before treatment). **(B)** Heatmap showing the expression of each cluster in a total of 33 samples, including liver cancer patients and healthy donors. **(C)** t-SNE dot plot depicting protein expression in platelet samples from liver cancer patients and healthy donors. The color bar ranges from blue to red, with red indicating higher expression levels. **(D)** Box plot comparing the differences in platelets between liver cancer patients and healthy donors in different platelet subpopulations. **(E)** Heatmap showing the expression patterns of platelet subpopulation markers, with the color bar ranging from blue to red, indicating higher expression levels in red. **(F)** Stacked bar chart representing the proportion of platelet subpopulations in each sample group.

First, we conducted a clustering analysis of platelet samples from the plasma of 23 liver cancer patients before treatment and 10 healthy donors. We identified 11 distinct platelet subpopulations (**Figures 1A, B**). Independent t-SNE heatmaps reflected the relative relationships between proteins and platelet subpopulations (**Figure 1C**). Comparing platelet subpopulations between liver cancer patients and healthy donors, we found that the proportions of Cluster 7 and Cluster 11 were lower in liver cancer patients than in healthy donors (**Figure 1D**). The heatmap displaying protein expression across platelet subpopulations revealed that Cluster 7 had the highest expression of CD107a among all subpopulations, and its expression was almost exclusively restricted to Cluster 7, with little to no expression in other subpopulations. Similarly, Cluster 11 exhibited the highest expression of CD62P, although other subpopulations also expressed CD62P, but at lower levels than Cluster 11. Notably, compared to other CD62P^+^ subpopulations, Cluster 11 showed lower CD29 expression, which can serve as a distinguishing feature for differentiating Cluster 11 from other CD62P^+^ subpopulations (**Figures 1E**). The distribution of platelet subpopulations in different cases was described using stacked bar charts (**Figures 1F**). Thus, we concluded that the state and function of platelets in the plasma of liver cancer patients differ from those in healthy donors. To further explore these differences, we grouped the pre-treatment samples of liver cancer patients according to treatment efficacy to investigate the variations in platelets.

### CD29+ platelet subpopulation is associated with poor response to combined therapy in liver cancer

**Figure 2:**
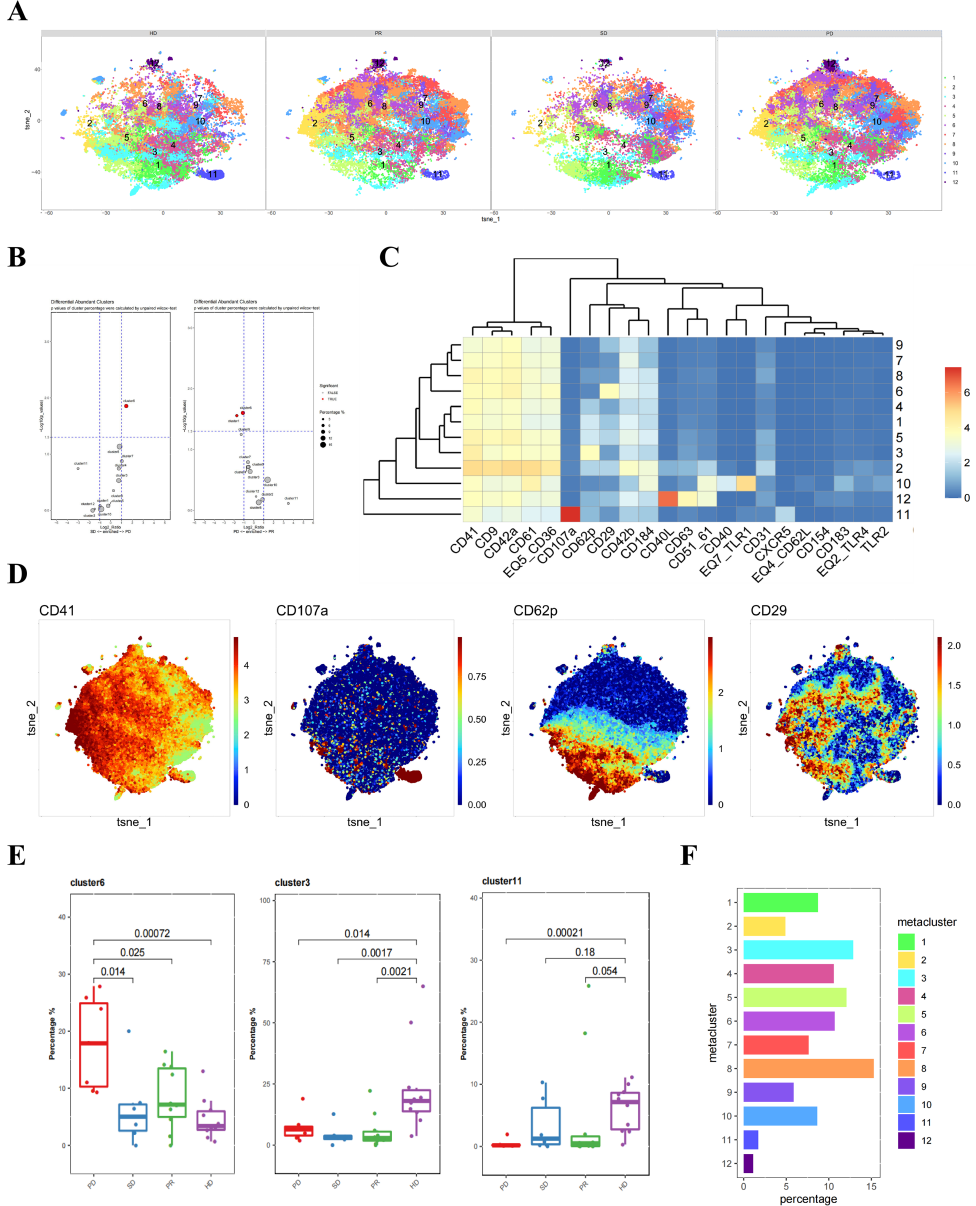
Differences in platelets between untreated liver cancer patients and healthy donors with different prognoses. **(A)** CyTOF analysis showing the differences in platelets between untreated liver cancer patients with different prognoses and healthy donors. t-SNE clustering based on protein expression levels in different groups (HD group for healthy donors, PD group for progressive disease, SD group for stable disease, PR group for partial response). **(B)** Volcano plot showing the differences in clusters among different groups of liver cancer patients. p-values of cluster percentages were calculated by unpaired Wilcox test. **(C)** Heatmap showing the expression patterns of platelet subpopulation markers, with the color bar ranging from blue to red, indicating higher expression levels in red. **(D)** Heatmap showing the expression of each cluster in a total of 33 samples, including different groups of liver cancer patients and healthy donors. **(E)** Box plot comparing the differences in platelets between liver cancer patients and healthy donors in different platelet subpopulations. **(F)** Bar chart showing the proportion of platelet subpopulations in each sample group.

Next, we classified the pre-treatment platelet samples from liver cancer patients into three groups based on clinical outcomes: PD (progressive disease), SD (stable disease), and PR (partial response). These samples, along with those from healthy donors, were subjected to clustering analysis, identifying a total of 12 platelet subpopulations (**Figure 2A**). Using an unpaired Wilcox test to calculate the p-values for cluster percentages, we found significant differences in cluster 6 when comparing the PD group to both the SD and PR groups (**Figure 2B**). Platelets in cluster 6 exhibited higher CD29 expression compared to other clusters, indicating that CD29+ platelet subpopulations might be related to future treatment outcomes (**Figure 2C**).

Additionally, independent t-SNE heatmaps reflected the relative relationships between proteins and platelet subpopulations (**Figure 2D**). The previously identified CD107a+ and CD62P+CD29- platelet subpopulations corresponded to clusters 11 and 3 in this clustering. Distinct differences were observed between liver cancer patient groups and healthy donors in the CD62P+CD29- platelet cluster 3. A similar trend was seen in the CD107a+ platelet cluster 11, although the distinction was only significant between the PD and HD groups (**Figure 2E**). The proportion of each platelet subpopulation was visually represented using bar charts (**Figure 2F**).

In conclusion, grouping liver cancer patients’ pre-treatment plasma samples according to treatment efficacy revealed significant differences between the PD group and other groups, primarily in the CD29+ platelet subpopulations. Therefore, this subpopulation may be associated with poor prognosis in liver cancer patients.

### Differences in CD29+ platelet subpopulation before and after combined therapy in liver cancer

**Figure 3:**
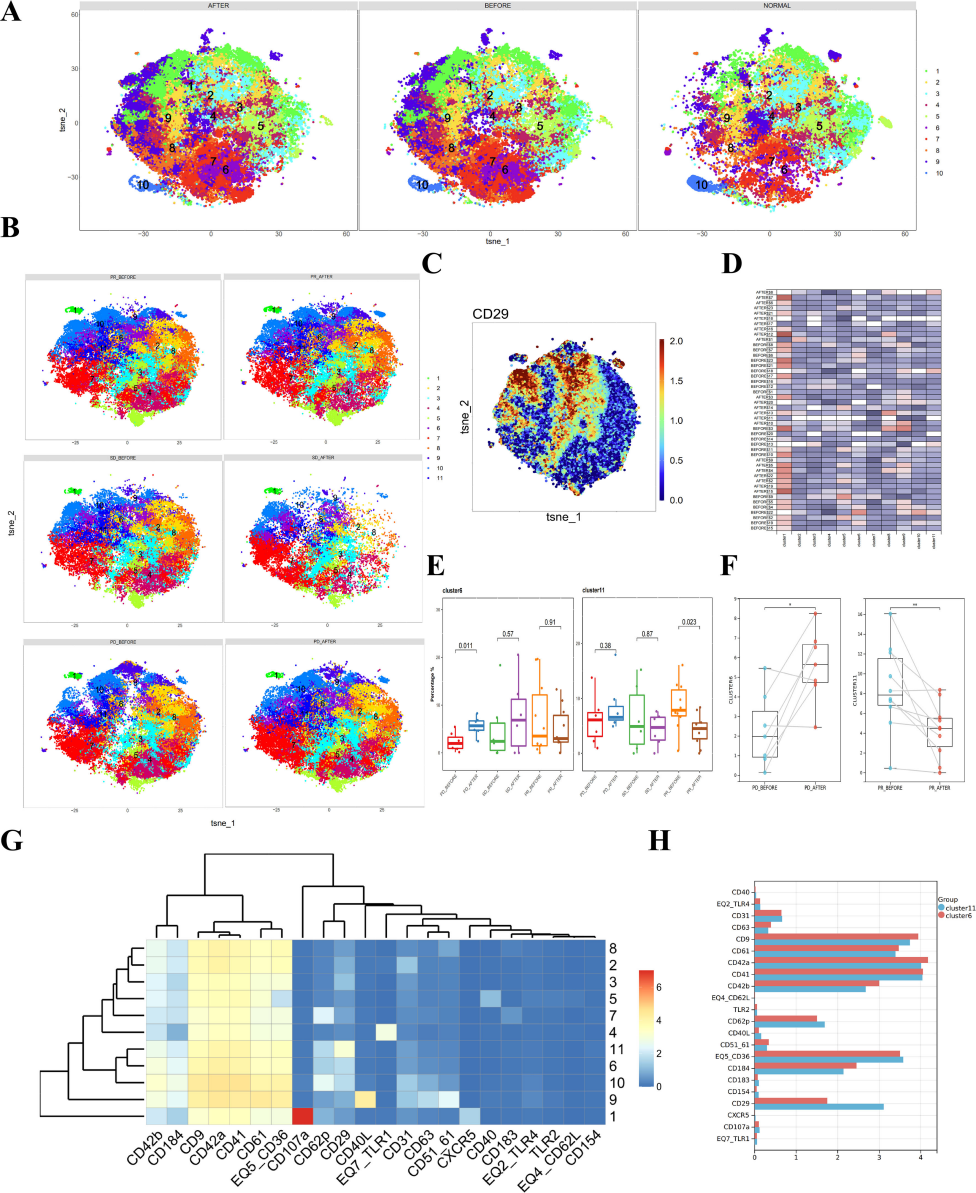
Differences in platelets before and after combined immunotherapy and anti-angiogenic therapy in liver cancer patients with different prognoses. **(A)** CyTOF analysis showing the differences in platelets between liver cancer patients and healthy donors. t-SNE clustering based on protein expression levels in different groups (NORMAL group for healthy donors, BEFORE group for liver cancer patients before treatment, AFTER group for liver cancer patients after treatment). **(B)** CyTOF analysis showing the differences in platelets between liver cancer patients with different prognoses before and after treatment and healthy donors. t-SNE clustering based on protein expression levels in different groups (PD_BEFORE group for progressive disease before treatment, PD_AFTER group for progressive disease after treatment, SD_BEFORE group for stable disease before treatment, SD_AFTER group for stable disease after treatment, PR_BEFORE group for partial response before treatment, PR_AFTER group for partial response after treatment). **(C)** Heatmap showing the expression patterns of CD29 in platelet subpopulations, with the color bar ranging from blue to red, indicating higher expression levels in blue. **(D)** Heatmap showing the expression of each cluster in a total of 33 samples, including liver cancer patients and healthy donors. **(E)** Box plot comparing the differences in platelet subpopulations between liver cancer patients and healthy donors. **(F)** Box plot showing paired differential analysis of platelet subpopulations in each liver cancer patient group before and after treatment. (*p < 0.05, **p < 0.01). **(G)** Heatmap showing the expression patterns of platelet subpopulation markers, with the color bar ranging from blue to red, indicating higher expression levels in red. **(H)** Multiple bar charts analyzing the expression levels of various markers in platelet subpopulations.

To determine whether combined immunotherapy and anti-angiogenic therapy affect platelet subpopulations in patients’ plasma, we analyzed platelet samples from 23 liver cancer patients before and after treatment. A direct comparison of platelet subpopulations in pre- and post-treatment plasma showed no significant differences (**Figure 3A**).

Subsequently, we separately analyzed platelet samples before and after treatment in the PD, PR, and SD groups, comparing changes in platelet subpopulations. Clustering analysis of platelets before and after treatment in liver cancer patients identified 11 subpopulations (**Figure 3B**). Given the previously observed differences in CD29+ platelet subpopulations among different patient groups, we focused on these subpopulations. t-SNE heatmaps reflected the relative relationships between CD29 and platelet subpopulations (**Figure 3C**). The expression of each subpopulation in individual samples was displayed in heatmaps (**Figure 3D**).

Comparing platelet changes before and after treatment in each group, we found that cluster 6 increased in the PD group, while cluster 11 decreased in the PR group (**Figure 3E**). Paired analysis of pre- and post-treatment platelet samples showed statistically significant differences (**Figure 3F**). Notably, both clusters 6 and 11 were CD29+ (**Figure 3G**). Analyzing the differences in protein expression levels within these two subpopulations, we found that the primary difference between the two CD29+ platelet subpopulations was the expression level of CD29, with cluster 11 showing higher CD29 expression (**Figure 3H**). Therefore, we suggest that CD29+ platelet subpopulations play a significant role in the progression and treatment of liver cancer.

## Discussion

Combined immunotherapy and targeted therapy has become the first-line treatment option for liver cancer, showing good anticancer effects in most patients. However, some patients still experience poor efficacy. Therefore, there is a lack of biomarkers to assess the prognosis of liver cancer patients undergoing combined immunotherapy and targeted therapy.

Platelets, derived from megakaryocytes, are crucial components of blood, primarily involved in hemostasis and coagulation (19). They can also regulate tumor nutrition supply by altering the angiogenesis around tumors (20). Additionally, platelets play a role in immune regulation (21). Studies have shown that platelets can alter the tumor immune microenvironment by inhibiting T cells, thereby suppressing the immune response against tumors (22). They can also support immune evasion by participating in communication between immune cells (23). Therefore, the relationship between platelets and tumors is currently a key area of research.

Despite the abundance of platelets in plasma and their ease of preparation and isolation, little research has explored the heterogeneity of platelet subpopulations in liver cancer. In this study, we labeled platelets in plasma with 22 metal-conjugated antibodies and used CyTOF technology to differentiate and analyze these platelets based on their surface antigen expression. We identified CD107a+ and CD62p+ platelet subpopulations in liver cancer patients, which were significantly downregulated compared to healthy individuals.

CD107a, a lysosome-associated membrane protein-1 (LAMP-1), translocates to the cell surface during platelet activation. Its expression is a marker of platelet degranulation, reflecting the activation and granule release state of platelets (24). CD62p, also known as P-selectin, moves from α-granules to the platelet surface upon activation, indicating platelet activation and degranulation (25). Activated platelets change shape and release internal granules to perform their functions (25, 26). Our study results showed that the subpopulations of CD107a+ and CD62p+ platelets were reduced in untreated liver cancer patients. This may indicate a decrease in platelet activation and a weakened platelet function in liver cancer.

We further grouped patients based on clinical efficacy and found that CD29+ platelets were significantly higher in the progressive disease group compared to other groups, suggesting a correlation with clinical outcomes. CD29, also known as β1 integrin, is a receptor in the integrin family that promotes platelet activation, adhesion, and participation in angiogenesis (27). In cancer, CD29-high platelets can enhance tumor angiogenesis, providing more nutrients and oxygen to tumors, thereby promoting tumor growth and metastasis (28). CD29 plays a crucial role in cell adhesion, signal transduction, angiogenesis, and tumor metastasis. Drugs targeting β1 integrin, such as Volociximab, are currently under investigation for their antitumor effects (29). In our results, the high expression of the CD29+ platelet subpopulation in the PD group may be the reason for the poor therapeutic efficacy in these patients. The elevated levels of CD29+ platelet subpopulation could serve as a biomarker for predicting poor efficacy of combined immunotherapy and targeted therapy in liver cancer.

Finally, we compared the differences in platelet subpopulations before and after combined immunotherapy and targeted therapy in each patient group. We found that CD29+ platelets could be further divided into two subpopulations based on CD29 expression levels. The higher CD29-expressing subpopulation decreased with tumor regression in the partial response (PR) group, while the lower CD29-expressing subpopulation increased with tumor progression in the PD group. This indicates that CD29+ platelet subpopulations play a significant role in tumor progression. The potential to improve the efficacy of combined immunotherapy and targeted therapy in liver cancer by modulating CD29+ platelets warrants further investigation.

In summary, this study explored the differences in platelet subpopulations in liver cancer patients undergoing combined immunotherapy and targeted therapy. Using CyTOF analysis, we found that the reduction of CD107a+ and CD62p+ platelet subpopulations in liver cancer patients might be related to the tumor immune environment and vascular status. Moreover, CD29+ platelets were associated with poor treatment outcomes, fluctuating with tumor regression and progression. Our results suggest that CD29+ platelets could serve as a prognostic indicator for the efficacy of combined immunotherapy and targeted therapy in liver cancer. Future research should investigate the potential of targeting CD29 to improve treatment outcomes in liver cancer patients.

## Data Availability

The original contributions presented in the study are included in the article/**Supplementary Materials**. Further inquiries can be directed to the corresponding authors.
