# Supplementary material for: CyTOF reveals platelet subtype changes predicting the efficacy of combined immunotherapy and targeted therapy in liver cancer
Source: Front Immunol. 2025 May 27;16:1538652. doi: 10.3389/fimmu.2025.1538652 (PMC12149196; doi:10.3389/fimmu.2025.1538652)
Supplement: Supplementary file 2 [file Table1.docx]

**Supplementary Table 1. Antibodies panel designed for CyTOF**

| Mass | Antigen | Antibody clone | Source |
| --- | --- | --- | --- |
| 141 | CD40 | 5C3 | Fluidigm |
| 142 | TLR4 | HTA125 | Biolegend |
| 144 | CD31 | WM59 | Fluidigm |
| 146 | CD63 | 2585J | R&D |
| 147 | CD9 | SN4 C3-3A2 | Fluidigm |
| 148 | CD61 | VI-PL2 | Biolegend |
| 149 | CD42a | ALMA.16 | BD |
| 150 | CD41 | HIP8 | Biolegend |
| 152 | CD42b | HIP1 | Biolegend |
| 153 | CD62L | DREG-56 | Fluidigm |
| 155 | TLR2 | W15145C | Biolegend |
| 159 | CD62p | AK4 | Biolegend |
| 162 | CD40L | CD40LG/2761 | abcam |
| 164 | CD51_61 | 23C6 | Biolegend |
| 165 | CD36 | 5-271 | Biolegend |
| 166 | CD184 | 12G5 | Biolegend |
| 167 | CD183 | G025H7 | Fluidigm |
| 168 | CD154 | 24-31 | Fluidigm |
| 169 | CD29 | HUTS-21 | BD |
| 171 | CXCR5 | RF8B2 | Fluidigm |
| 172 | CD107a | H4A3 | Fluidigm |
| 176 | TLR1 | [TLR1.136](https://www.biolegend.com/en-us/search-results?Clone=TLR1.136) | Biolegend |

**Supplementary Table 2: Patients Information**

|  | Total | Group PR | Group SD | Group PD |
| --- | --- | --- | --- | --- |
| Gender-n(%) |  |  |  |  |
| Male | 21(91.3%) | 10(100%) | 6(100%) | 5(71.4%) |
| Female | 2(8.7%) | 0 | 0 | 2(28.6%) |
| Age（years old） | 54.0±8.7 | 55.3±7.5 | 53.3±12.4 | 51.1±7.2 |
| ECOG PS-n(%) |  |  |  |  |
| 0 | 7(30.4%) | 4(40) | 1(16.7) | 2(28.6%) |
| 1 | 16(69.6%) | 6(60) | 5(83.3) | 5(71.4%) |
| AFP(ng/ml)-n(%) |  |  |  |  |
| < 400 | 13(56.5%) | 7(70%) | 3(50%) | 3(42.9%) |
| ≥ 400 | 10(43.5%) | 3(30%) | 3(50%) | 4(57.1%) |
| Child-Pugh Class-n(%) |  |  |  |  |
| A | 12(52.2%) | 4(40%) | 4(66.7%) | 4(57.1%) |
| B | 11(47.8%) | 6(60%) | 2(33.3%) | 3(42.9%) |
| Macrovascular invasion-n(%) |  |  |  |  |
| No | 8(34.8%) | 5(50%) | 6(60%) | 3(42.9%) |
| Yes | 15(65.2%) | 5(50%) | 0 | 4(57.1%) |
| Distant metastasis-n(%) |  |  |  |  |
| No | 14(60.9%) | 8(80%) | 3(50%) | 4(57.1%) |
| Yes | 9(39.1%) | 2(20%) | 3(50%) | 3(42.9%) |

**Supplementary Table 3: Patient Grouping and Grouping Criteria**

| **Patient ID** | **Baseline** | **Post-treatment assessment** | **Group** | **Grouping Criteria** |
| --- | --- | --- | --- | --- |
| **1** | Intrahepatic target lesion: 1: Long diameter 19mm; 2: Long diameter: 23mm; | Intrahepatic target lesion disappeared | PR | Target lesion diameter reduction >30% |
| **2** | Intrahepatic target lesion: 1: Long diameter 46mm; 2: Long diameter 39mm | Multiple new intrahepatic lesions, largest diameter 22mm. | PD | New lesions |
| **3** | Intrahepatic target lesion: Long diameter 63mm; Thoracic spine target lesion: Long diameter 34mm | Intrahepatic target lesion: Long diameter 43mm; Thoracic spine target lesion: Long diameter 28mm | SD | \| Target lesion diameter reduction <30% \| \| --- \| |
| **4** | Intrahepatic target lesion: 1: Long diameter 81mm; 2: Long diameter 67mm | Intrahepatic target lesion: 1: Long diameter 120mm; 2: Long diameter 92mm. | PD | Target lesion diameter increase >20% |
| **5** | Intrahepatic target lesion: 1: Long diameter 165mm; 2: Long diameter 21mm. | Intrahepatic target lesion: 1: Long diameter 213mm; 2: 26mm; | PD | Target lesion diameter increase >20% |
| **6** | Intrahepatic target lesion long diameter 146mm | Intrahepatic target lesion: Residual lesion 1: Long diameter 47mm; Residual lesion 2: Long diameter 43mm | PR | Target lesion diameter reduction >30% |
| **7** | Intrahepatic target lesion long diameter 80mm; | Intrahepatic target lesion long diameter 55mm | PR | Target lesion diameter reduction >30% |
| **8** | Intrahepatic target lesion: 1: Long diameter: 33mm; 2: Long diameter 25mm; Hepatogastric lymph node: Short diameter 15mm; | Intrahepatic target lesion: 1: Long diameter: 29mm; 2: Long diameter 0mm; Hepatogastric lymph node: Short diameter 13mm; | PR | Target lesion diameter reduction >30% |
| **9** | Intrahepatic target lesion: Long diameter 204mm; | New multiple metastatic lesions in the right lobe of the liver, largest diameter >10mm | PD | New lesions |
| **10** | Intrahepatic target lesion: Long diameter 168mm; | Intrahepatic target lesion: Long diameter 129mm; | SD | \| Target lesion diameter reduction <30% \| \| --- \| |
| **11** | Intrahepatic target lesion: Long diameter 103mm; | Intrahepatic target lesion: Long diameter 105mm; | SD | Target lesion diameter increase <20% |
| **12** | Intrahepatic target lesion long diameter 105mm | Intrahepatic target lesion long diameter 24mm | PR | Target lesion diameter reduction >30% |
| **13** | Intrahepatic target lesion: Long diameter 196mm; | New intrahepatic lesion: Long diameter 45mm | SD | \| Target lesion diameter reduction <30% \| \| --- \| |
| **14** | Intrahepatic target lesion long diameter: 134mm; Pulmonary target lesion long diameter: 14mm; Adrenal target lesion: Long diameter 30mm | Intrahepatic target lesion: Long diameter 105mm; Pulmonary target lesion: Long diameter 8mm; Adrenal target lesion: Long diameter 13mm | SD | \| Target lesion diameter reduction <30% \| \| --- \| |
| **15** | Intrahepatic target lesion: 1: 3735; 2: 3532mm | New intrahepatic lesion: Long diameter 10mm | PD | New lesions |
| **16** | Intrahepatic target lesion long diameter 20mm; | Intrahepatic target lesion disappeared | PR | Target lesion diameter reduction >30% |
| **17** | Intrahepatic target lesion long diameter 130mm | Intrahepatic target lesion long diameter 50mm | PR | Target lesion diameter reduction >30% |
| **18** | 1. Intrahepatic target lesion long diameter 88mm: 2. Lymph node 1 short diameter: 25mm, Lymph node 2 short diameter 22mm; | 1. Intrahepatic target lesion long diameter: 57mm; 2. Lymph node 1 short diameter: 13mm; Lymph node 2 short diameter: 12mm; | PR | Target lesion diameter reduction >30% |
| **19** | Intrahepatic target lesion: Long diameter 104mm | Intrahepatic target lesion: Long diameter 98mm; New tumor thrombus in the inferior vena cava. | PD | New lesions |
| **20** | Intrahepatic target lesion: Long diameter 140mm; | Intrahepatic target lesion: Long diameter 148mm | SD | Target lesion diameter increase <20% |
| **21** | Liver cancer target lesion long diameter 14mm; | Intrahepatic target lesion disappeared | PR | Target lesion diameter reduction >30% |
| **22** | Left lung target lesion: Long diameter 21mm; Right lung target lesion long diameter 25mm | New pulmonary lesion: Long diameter 16mm | PD | New lesions |
| **23** | Liver cancer target lesion long diameter 10mm; | Intrahepatic target lesion disappeared | PR | Target lesion diameter reduction >30% |
